# GIN'n'CIN hypothesis of brain aging: deciphering the role of somatic genetic instabilities and neural aneuploidy during ontogeny

**DOI:** 10.1186/1755-8166-2-23

**Published:** 2009-11-25

**Authors:** Yuri B Yurov, Svetlana G Vorsanova, Ivan Y Iourov

**Affiliations:** 1 National Research Center of Mental Health, Russian Academy of Medical Sciences, Moscow 119152, Russia; 2 Institute of Pediatrics and Children Surgery, Rosmedtechnologii, Moscow, 127412, Russia

## Abstract

Genomic instability (GIN) and chromosome instability (CIN) are two closely related ways to produce a variety of pathogenic conditions, i.e. cancer, neurodegeneration, chromosomal and genomic diseases. The GIN and CIN manifestation that possesses the most appreciable impact on cell physiology and viability is aneuploidy. The latter has been consistently shown to be associated with aging. Classically, it has been considered that a failure of mitotic machinery leads to aneuploidy acquiring throughout aging in dividing cells. Paradoxically, this model is inapplicable for the human brain, which is composed of post-mitotic cells persisting throughout the lifetime. To solve this paradox, we have focused on mosaic neural aneuploidy, a remarkable biomarker of GIN and CIN in the normal and diseased brain (i.e. Alzheimer's disease and ataxia-telangiectasia). Looking through the available data on genomic variations in the developing and adult human central nervous system, we were able to propose a hypothesis suggesting that neural aneuploidy produced during early brain development plays a crucial role of genetic determinant of aging in the healthy and diseased brain.

## Introduction

Aneuploidy has been consistently shown to be associated with aging [1-5]. However, there is no consensus on how aneulpoidization and aging are interconnected. Several lines of evidences indicate that increasing rate of mitotic errors in late ontogeny can be a mechanism for chromosome gains and losses in aging tissues [6]. This corresponds to data on aneuploidy in human tissues composed of mitotic cells [2-4], but is inapplicable to post-mitotic cells. In this context, the human brain is probably the most remarkable example of a tissue populated by almost exclusively post-mitotic cells that are not suggested to undergo mitotic division [7,8]. One can assume a brain aging mechanism without aneuploidization, but this is not in accordance with molecular neurocytogenetic observations of the adult human brain demonstrating the presence of aneuploid cells [9-14]. The dilemma might be solved by addressing adult neurogenesis research that has depicted the possibility of the adult mammalian brain to generate new neuronal cells [15]. However, these data is unable to provide complete explanation of aging-related changes within the content of chromosomal DNA in the normal and diseased human brain, which indicates aneuploidy association with brain aging phenotypes [7,9-14,16-20]. Nonetheless, the brain is aging!

At the turn of the last century, there were no less than 300 theories for explanation of aging phenomenon [21]. It is apparent, that none of these hypotheses would give the ultimate explanation of such a complex process. Nevertheless, some theories, especially those aimed to gather different data from several biomedical areas, appear to be more "close to the reality". Among these models, there is one based on cellular/molecular parallels between processes occurring in senescent and cancer cells [22]. The commonest pattern of molecular changes observed in both aging and cancer cell is genomic instability (GIN). This can manifest at different molecular and microscopic levels: single nucleotides, nucleotide repeat size variations, loss/gains of genomic regions, loss of chromosome structural integrity, structural chromosome abnormalities, aneuploidy or polyploidy. Analysis of cancers and aged tissues suggests that instability of the nuclear genome is more frequently involves changes occurring at chromosomal level. The latter, in some instances, may manifest as chromosome instability (CIN) [7,12,17,20,22-26]. Pathological consequences of GIN or CIN usually become appreciable *via *its accumulation throughout ontogeny [7,12,22,23]. In other words, phenotypic consequences of GIN and CIN are not necessarily immediate. This fact is intriguing in the light of ontogenetic variations of aneuploidy rates in the normal human brain and presence of GIN/CIN in the diseased brain (especially, in cases of individuals suffering from accelerated aging diseases) [5,7-14,16-20,27,28]. Summarizing current data on somatic genome behavior in brain, cancer and aging tissues, it is hard to avoid making some parallels. Similar phenomena (GIN and CIN) occurring in these biological systems are unlikely to be a simple coincidence. This has led us to an attempt of gathering current knowledge about GIN and CIN in the human brain to achieve an integrated view of the interplay between aneuploidy and aging in the human brain. As a result, a GIN'n'CIN (GIN and CIN) hypothesis connecting data on these phenomena and brain aging was proposed.

This hypothesis was inspired by recent advances in chromosome research of the human brain or molecular neurocytogenetics of individuals with/without brain diseases. Among these, there were some associated with accelerated or abnormal aging. Furthermore, appreciable ontogenetic variations of CIN levels in the human brain were noticed. Therefore, to make a clear presentation of our hypothesis, we found pertinent first to provide a description of brain-specific GIN/CIN occurrence at different ontogeny stages and in brain diseases, especially when accompanied by accelerated aging.

## Aneuploidy in the human brain: cell fate and ontogeny

It has been long debated whether the mammalian brain is populated by cells with abnormal chromosome numbers (polyploid cells) [7,29-31]. However, these observations were not definitive, inasmuch as there were not a technique for direct analysis of chromosomes in non-dividing interphase cells [7,9,12,16]. The breakthrough in developing new molecular cytogenetic approaches towards analyses of interphase chromosomes has allowed direct visualization of individual chromosomes in the human brain. The latter was associated with the introduction of multiprobe fluorescence *in situ *hybridization (mFISH), quantitative fluorescence *in situ *hybridization (QFISH) and interphase chromosome-specific multicolor chromosome banding (ICS-MCB, the unique technique allowing analysis of whole interphase chromosomes in their integrity) [5,7,9-14,16-20,27,28,32-35]. As a result, a series of high-resolution studies targeted at definition of chromosome number variations in the normal brain were performed and elucidated aneuploidy to be the essential source for the genetic intercellular diversification. Table 1 gives an overview of molecular neurocytogenetic data on aneuploidy the normal human brain obtained by the aforementioned techniques. The human brain has exhibited relatively high rate of sporadic (stochastic) aneuploidy, taking into account its post-mitotic nature [7-11,14,19,20,27,28]. Thus, the developing human brain has been shown to possess about 30% of aneuploidy cells. This cell number is surprisingly close to the amount of cells cleared by programmed cell death throughout prenatal brain development [7,9,27,28]. Therefore, it was unsurprising that the adult human brain exhibited aneuploidy rates being almost exactly three-fold less than that in the developing human brain (Table 1) [7-14,19,20].

**Table 1:** Aneuploidy in the developing and normal adult human brain

	Rate of aneuploidy, % (per chromosome/whole genome)	Evaluation technique	References
Developing brain:			

direct evaluation	0.6-3.0/~28	mFISH	[9]

	1.25-1.45/30-35	mFISH/QFISH ICS-MCB	[28]

after *in vitro *cultivation	1.2-7.0/72.4	mFISH	[9,27]

Adult brain:			

	0.1-0.8/~9	mFISH	[9,11]

	0.2-2/10	ICS-MCB	[10]

	0.2-0.7/~10	mFISH/QFISH ICS-MCB	[14]

Abbreviations: mFISH -- multiprobe fluorescence *in situ *hybridization; QFISH -- quantitative fluorescence *in situ *hybridization; ICS-MCB -- interphase chromosome-specific multicolor chromosome banding.

These ontogenetic variations of neuronal genome are undoubtedly determined by the fate of aneuploid brain cells [7]. There have been provided extensive data on genetic and epigenetic phenomena that influence neuronal cell fate [8,36]. However, effects of aneuploidy were not experimentally addressed in this context.

**Figure 1:**
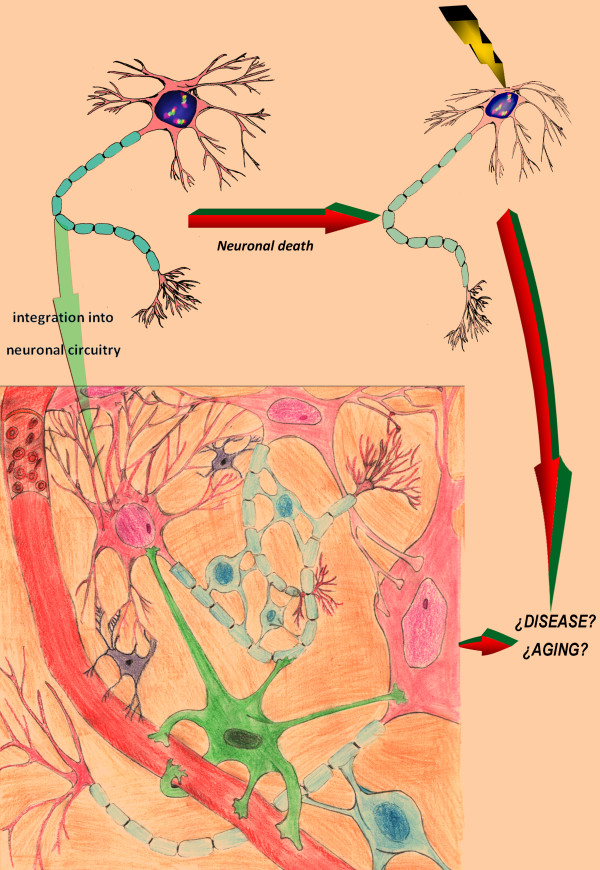
**The fate of an aneuploid neuron**. It seems that there are two most probable ways. Firstly, aneuploid neuron can be integrated into neuronal circuitry. This has the potential to produce disease phenotype (through affecting all the elements of the circuitry *via *synaptic activity of aneuploid neuron) or this could be a probable mechanism of aging; alternatively, if aneuploidy rates are relatively low, integration of aneuploid neuron into neuronal circuitry is suggested to be a mechanism for neuronal diversity. Secondly, aneuploid neuron can be subjected to neuronal cell death. The latter could be associated with large-scale neuronal cell clearance that has potential to lead to brain diseases or, alternatively, to be an aging mechanism.

Nevertheless, some considerations can be made according to current knowledge of aneuploidy effects and associations in other somatic tissues or accelerated aging disorders [5,7,14,17,20,28,37]. Figure 1 demonstrates the essential ways, according which the fate of an aneuploid neuron can develop. Regardless difficulties of making definite conclusions concerning relationship between neuronal cell fate and human brain diseases, it is to recognize that the failure of neuronal homeostasis and excessive loss of neurons due to aneuploidy or integration of aneuploid neurons into neuronal circuitry should negatively affect the functioning of the human brain. In any case, the presence of aneuploid neurons is rather pathological sign than a normal condition. Therefore, extensive aneuploidization of neural cells is a highly probable mechanism for brain diseases. Additionally, aneuploid neurons could be implicated in aging processes. Without direct experimental proofs, this can be tested addressing brain diseases associated with accelerated aging.

## Diseases of accelerated aging due to unstable genome: a neurocytogenetic overview

Currently, there are no fewer than 25 accelerated aging or progeroid diseases associated with genetic instabilities, including monogenic and chromosomal diseases [22,38-40]. The most striking common features that almost all these disorders possess is referred to CIN/GIN and cancer susceptibility. A number of these diseases are associated with neurodegeneration. Usually, such diseases are caused by mutations in genes involved in maintenance of the genome integrity or chromosome structure [38,40].

Aneuploidy has long been noticed to produce senescent cellular phenotype [41]. Therefore, it is not surprising that the most common aneuploidy syndrome (Down syndrome or trisomy of chromosome 21) is associated with premature aging and neurodegeneration [22,38]. Down syndrome is uncommonly associated with CIN or chromosomal mosaicism. It is suggested that over 95% of cases are associated with trisomy 21 affecting all the cells of the organism (including brain cells) [42,43]. However, a subtype of this syndrome termed mosaic trisomy 21 exhibiting milder symptoms of Down syndrome does exist [44]. Additional chromosome 21 has profound effects on brain functioning and morphology in individuals with Down syndrome [37]. The latter provides for a speculative conclusion that aneuploidy could cause premature brain aging, but it does not exclude the need of an experimental proof, which has not been, as yet, provided [7,12]. Furthermore, mosaic trisomy 21 detected in fetal ovarian tissues was found to be a likely explanation for maternally derived aneuploidy in conceptuses of aged mothers [45]. Nonetheless, neither CIN nor GIN has been evaluated in the Down syndrome brain. Probably, it would not exhibit high rate of CIN nor GIN, inasmuch as trisomy 21 being an already unbalanced chromosome complement has not been described to produce further GIN/CIN in human somatic cells. However, related studies will certainly give hints to aneuploidization theory of aging.

Regarding aneuploidy association with aging, it is to mention a hypothesis suggesting common origins of Down syndrome and Alzheimer's disease. More precisely, it has been proposed that Alzheimer's disease brain pathology is likely to result from mosaic chromosome 21 aneuploidy [46]. Subsequent indirect evaluations performed on different Alzheimer's disease models have formed a strong experimental basis for this hypothesis [47,48]. Moreover, low-level mosaic aneuploidy of chromosome 21 is repeatedly observed in Down syndrome offspring's mothers, who are susceptible to Alzheimer's disease, as well [49]. Thus, brain-confined aneuploidy has appeared to be involved in Alzheimer's disease pathogenesis [7]. To test these assumptions, aneuploidy was monitored in the cerebral cortex of the Alzheimer's disease brain. The study has shown a dramatic 10-fold increase of chromosome 21-specific aneuploidy and a conclusion that somatic mosaic aneuploidy of chromosome 21 does play a critical role in Alzheimer's disease pathogenesis has been made [14]. Together, molecular neurocytogenetic analyses of Alzheimer's disease suggest aneuploidy to be involved in brain aging.

Another extraordinary example of a progeroid disease associated with brain dysfunction and CIN is ataxia-telangiectasia [38,40]. This autosomal recessive disease is associated with mutations in *ATM *(a gene involved in genome maintenance as well as cell cycle regulation and apoptotic pathways [50]) that are responsible for multilateral CIN in affected individuals [20]. Ataxia-telangiectasia is characterized by targeted cerebellar neurodegeneration, oculocutaneous telangiectasia, cancer predisposition, radiosensitivity, immunodeficiency and progeroid features [50]. Clinical and molecular studies of this disease have brought tremendous advances in molecular and cell biology of cell cycle, cancerization, immunodeficiency and aging as well as neurobiology of human disease [20,50]. The analysis of ataxia-telangiectasia for testing hypothetic associations between CIN and accelerated aging becomes even more actual taking into account appreciable age-dependant changes in functioning of DNA repair machinery [51]. This disease is also featured by a paradox that refers to neurodegenerative processes occurring exclusively in the cerebellum (targeted cerebellar degeneration), while other brain areas are significantly less affected [52]. The solution of this paradox has been provided by molecular neurocytogenetic studies that have indicated selective confinement CIN/GIN to more severely affected brain areas (cerebellum) and aneuploidization of brain cells as essential mechanisms for ataxia-telangiectasia brain dysfunction [14,20]. Paradoxically, CIN was found to increase with age in the ataxia-telangiectasia brain [20]. Together, this data allows the speculation that increased CIN and GIN are involved in accelerated brain aging.

Table 2 summarizes available data on brain-specific GIN/CIN in accelerated aging diseases. Regardless moderate amount of such studies, a conclusion about strong association between GIN, CIN, and aneuploidy in the brain and accelerated brain aging is unavoidable. At least, it can be stated about genetic brain diseases that have been already studied.

**Table 2:** Molecular neurocytogenetic data on accelerated aging diseases

Disease	Neurocytogenetic findings	CIN	GIN	References
Down syndrome	Presumably 100% (?) of trisomy 21	NS*	NS*	[37,42,43]

Alzheimer's diseases (AD)	Chromosome 21-specific aneuploidy confined to degenerated brain areas	+	?	[14]

AD model (transfected human cells with presenelin 1 mutations)	Acquired chromosome missegregation causing aneuploidy	+	+	[46]

Ataxia telangiectasia	Aneuploidy; chromosome breakage producing additional rearranged chromosomes (partial aneuploidy) confined to the degenerated cerebellum	+	+	[14,20]

*NS -- not studied

Although other progeroid diseases exhibiting genetic instabilities are not primarily associated brain dysfunction [38,40], molecular neurocytogenetic studies of these syndromes can shed light onto mechanisms of accelerated brain aging. Since there are numerous parallels between aging and tumorigenesis, additional valuable data can be retrieved from the investigation of genetic diseases with brain cancer predisposition. It is noteworthy that Li-Fraumeni syndrome, which is associated with mutations in *p53*, a global transcription factor involved in cell cycle control, tumor suppression, apoptosis, etc., is a progeroid disease featured by a predisposition to the development of brain tumors [40,53]. Furthermore, a hypothesis that suggests the lack of clearance of neural cells affected by GIN and CIN during prenatal brain development to produce childhood brain tumors appear to be highly probable [54]. One can propose that similar processes occurring in late ontogeny are involved in rather non-malignant neural cell dysfunction and neurodegeneration than tumorigenesis. Nevertheless, all these issues require additional molecular neurocytogenetic studies.

### GIN'n'CIN hypothesis

**Figure 2:**
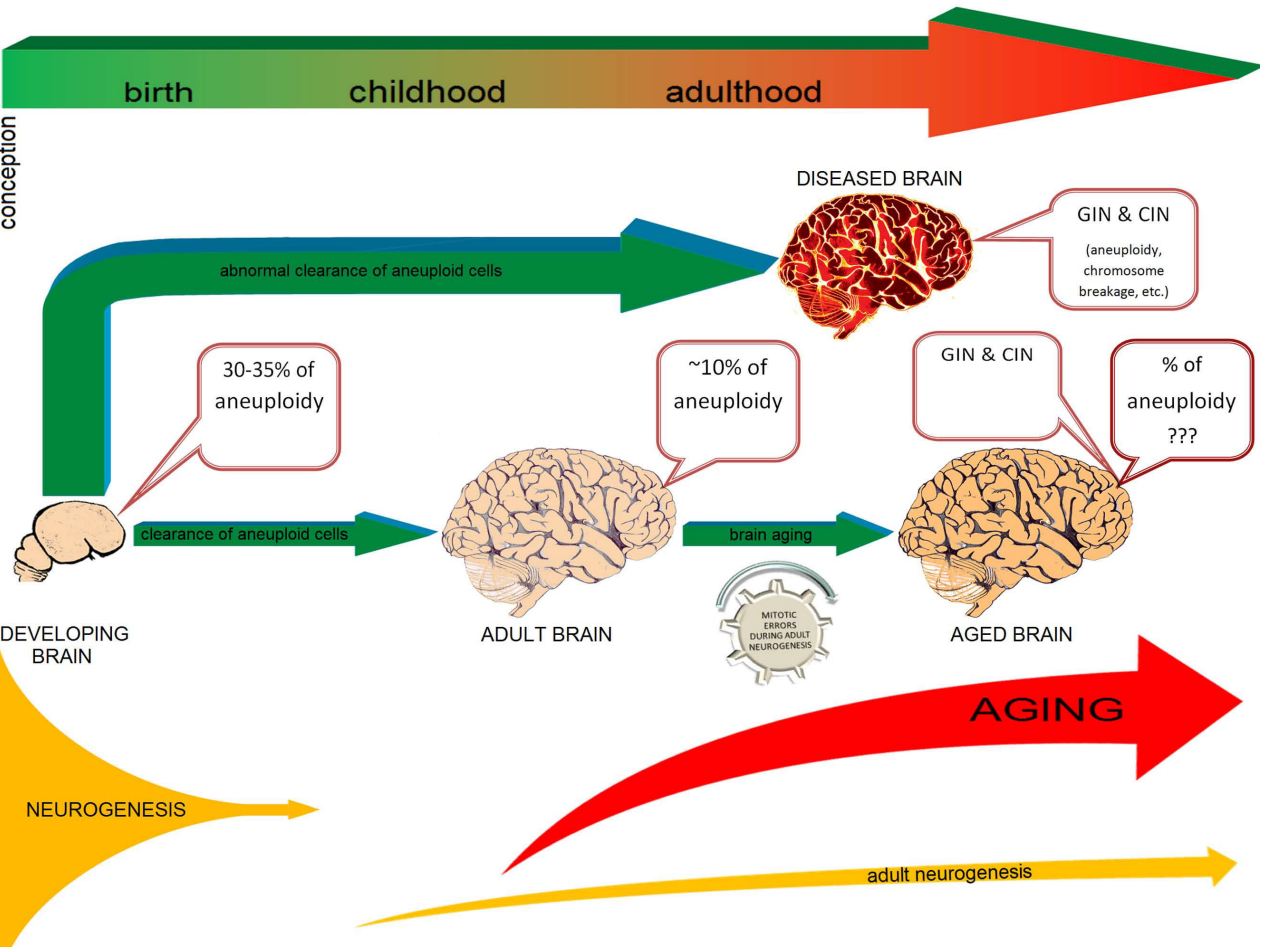
**The key points of the hypothesis**. The developing human brain (12-15 weeks' gestation) exhibit 30-35% of aneuploid cells [28], which are formed during neurogenesis (prenatal brain development). This process becomes exhausted soon after birth. At later developmental stage, adult neurogenesis starts, being, however, significantly less productive in terms of the amount of cells formed. Abnormal clearance of aneuploid cells leads to postnatal brain diseases, which are featured by GIN and CIN confined to the brain. Some of these diseases are associated with accelerated aging (i.e. Alzheimer's disease and ataxia-telangiectasia). Normal brain development leads to decrease of aneuploidy rates, which achieves averagely 10% [9,10,14]. The presence of aneuploid cells in the brain from the early prenatal development to the late ontogeny is hypothesized to give rise to GIN and CIN in the brain of elderly individuals. This is partially confirmed by analyzing controls in molecular neurocytogenetic studies of the diseased brain [5,7,12-14,16-20]. Mitotic errors during adult neurogenesis can also produce aneuploid cells throughout aging.

The hypothesis is based on the following aforementioned observations: (i) the developing human brain is prone to GIN/CIN and this leads to aneuploidization of 30-35% of fetal brain cells [28]; (ii) aneuploid cells are cleared throughout normal early brain development, this leads to decreasing of aneuploidy rate to approximately 10% after birth [9,10,14]; (iii) abnormal clearance of defected cells is associated with brain diseases, the majority of which are also characterized by accelerated (abnormal) aging and are featured by increased rates of GIN/CIN manifesting as aneuploidy or other types of genetic instabilities confined to the brain [5,7,12,14,16-20]; (iv) the brain has the potential to produce cell populations affected by GIN, CIN, or mosaic aneuploidy *via *mitotic errors during adult neurogenesis or age-related failures to repair damaged chromosomal DNA in neuronal cells [51]. Figure 2 schematically presents key points of our hypothesis. As one can see, there is a paradoxical feature of the human brain. More precisely, aneuploidization is initiated long before aging manifestations, leading to aneuploid cells production exclusively during early ontogeny. In other words, the most noticeable accumulation of aneuploid cells and propagation of CIN happen during the early development of the brain. In the first trimester of pregnancy, abnormal cells comprise almost exactly one third of the fetal brain population [28]. GIN is also shown to accumulate in neural/progenitor cells of the mammalian brain [55]. Developmental accumulation of GIN and CIN is similar or even more extensive as to that occurring in aging mitotic tissues [56]. However, when becomes composed preferentially of post-mitotic cells, the human brain exhibit a decrease of abnormal cell amount that, nonetheless, affects no fewer than 100 billion out of one trillion of neural cells [9,10,14]. Thus, at early ontogeny CIN/GIN rate makes a burst, then as neurogenesis slows down, aneuploidization does the same and the rate of CIN/GIN significantly decreases. If not decreased, both CIN and GIN lead to clinically distinguished brain diseases [7,14,16-20] (Figure 2). On the other hand, an appreciable proportion of aneuploid cells is present in the human brain being comparable to aneuploidy in other tissues composed of mitotic cells in elderly individuals [1-5,7,9-14,16-20]. This suggests that aneuploidy being an aging factor is generated in early development of the brain and persists throughout ontogeny. It remains uncertain whether aneuploidy rates vary because of aging. Does the aging program launch prior to birth? Is this a genetic program itself or is this a cascade of program errors due to developmental GIN/CIN? Although it sounds unusual, there are arguments for such speculations. Firstly, aneuploidy is associated with senescent cellular phenotype [7,37,38,40,41]. Secondly, 10% of aneuploid cells emerged during embryogenesis, among which at least a small proportion has from 10 to 10000 synapses (contacts with other brain cells), should possess an adverse effect on brain functioning [7]. Moreover, analysis of ataxia-telangiectasia murine models and the human ataxia-telangiectasia brain has demonstrated that failed clearance of aneuploid cells leads to premature aging phenotypes [20,57]. GIN and CIN leads to function losses or gains of RNAs and proteins (encoded by hundreds or thousands of genes localized in lost/gained chromosomes) involved in diverse cellular processes associated with aging. Together, it suggests that both GIN and CIN are phenomena involved in brain aging, but these genetic mechanisms are not the same as in "mitotic tissues". Since the brain cannot accumulate somatic chromosome mutations through mitotic divisions, it is difficult to solve this paradox. Here, we suggest aneuploidy remaining in the human brain after developmental cell clearance to be involved in cellular senescence processes. GIN/CIN initiates "molecular countdown" that begins long before phenotypic manifestations of brain aging in a manner of "delayed-action bomb". Therefore, mosaic aneuploidy acquired by the developing brain can be considered as an initiating element of the global genetic brain aging program. Probably, programmed cell death machinery that clears aneuploid cells in the developing brain ceases to function soon after birth. The latter appears to be convenient, because neurogenesis is unable to provide sufficient amount of cells to maintain proper brain cell amount that could be altered by the presence of aneuploid cells. Therefore, the persistence of aneuploid cells in the brain is rather a compromise between aneuploidization and exhausted neurogenesis than a required part of neuronal milieu. Senescent cell phenotypes generated by aneuploidy do not manifest immediately [41]. Therefore, mosaic aneuploidy can produce age-related changes in the brain after a period. Likewise, age-related cell loss begins to be noticeable in elderly individuals [58]. Moreover, neurodegenerative genetic brain diseases such as Alzheimer's disease, Down syndrome and ataxia-telangiectasia are featured by abnormal aging, aneuploidy and CIN/GIN affecting significant cell populations in the brain [14,20,42].

Does the adult human brain generate aneuploid cells? In this context, it is to refer to an important intention of current neuroscience, i.e. understanding of adult neurogenesis. Considering the rate of neuronal cells generated by this process [15], one can suggest its insignificant input as into confrontation with cell losses because of aging as into propagation of GIN/CIN in the aging brain. Nonetheless, our hypothesis does not completely exclude a contribution of adult neurogenesis to brain aging. We propose that mitotic errors during adult neurogenesis produce small amount of aneuploid brain cells, and this peculiarly contributes to GIN/CIN manifested as increased aneuploidy in late ontogeny. However, some comments on the hypothetic link between CIN/GIN and neurogenesis in the adult brain appear to be required. It is to notice that CIN (and, probably, GIN) is likely to affect non-neuronal (glial) cells in accelerated aging diseases [14,20]. Consequently, adult neurogenesis is unlikely to contribute significantly to age-related brain aneuploidization. One can immediately identify adult gliogenesis as the most probable candidate process for the normal and diseased brain aneuploidization, especially addressing current views suggesting this process to be more widespread than adult neurogenesis [59]. Nevertheless, adult gliogenesis has not been genetically studied in the same extent as adult neurogenesis was. Therefore, in the light of our hypothesis, making definite conclusions about the role of adult neurogenesis/gliogenesis in brain aging is incorrect. However, since mitotic machinery demonstrates failures because of aging [6], aneuploidization of cells generated through adult neurogenesis is possible.

According to our hypothesis, key points of brain aging mediated by GIN/CIN are as follows:

-- developmental genetic instabilities affecting fetal brain cells produces GIN and CIN that essentially manifests as an increase of aneuploidy rates;

-- programmed cell death diminish amount of cells affected by CIN/GIN during early brain development, but a significant proportion of aneuploid cells ultimately remains in the postnatal brain after birth and persists throughout ontogeny;

-- aneuploidy alters homeostasis of neuronal cells, generates senescent cellular phenotypes and, probably, promotes cell death; these processes begin to be apparent at phenotypic level in late ontogeny;

-- aneuploidy in the human brain is a unique aging-related phenomenon: it forms in early development, but acts as a genetic determinant of cellular senescence.

### Concluding remarks

One can argue whether there is a need of another aging theory. It is usually hard to find out a rationale of this multilateral process among different suggestions about the nature of cellular senescence leading to aging phenotypes. Such skepticism about theoretical considerations concerning aging hypotheses is unavoidable and suggests gathering of data from different areas of biomedicine to give an adequate explanation. Regardless several decades of cell senescence research, an integrated view on cellular/molecular basis of the aging is far from being complete [60]. Furthermore, commonly accepted hypotheses of aging (i.e. mitotic failure, telomere shortening, replicative stress etc.) cannot provide complete explanation of brain aging because of its "post-mitotic nature". Our hypothesis is not targeted at explanation of aging, as a whole, but is intended to define the meaning of causal relationship between aneuploidy and brain cell senescence. It accords well with observations of brain diseases featured by abnormal aging and aneuploidy effects on cellular homeostasis [14,20,37-42,47,48] as well as to theories of environmental effects associated with aging (i.e. effect of free radicals) [20], aging-cancer parallels [23,60], and somatic mutation accumulation [55,56]. Interestingly, the latter theory has been formulated exactly 50 years ago [61] and has been immediately challenged [62]. Evolutionary approaches dictated that maintenance of somatic genomes would not continue throughout the whole ontogeny leading, thereby, to age-dependent accumulation of somatic mutations [61]. Contrariwise, natural rate of somatic mutations was suggested to be low for producing an effect [62]. This discordance became more important in context of brain aging and, especially, after understanding of "post-mitotic nature" of the human brain. Ontogenetic periods have different somatic mutation rates (i.e. mitotic errors are more frequent during prenatal development than during postnatal periods) [7,17]. The present GIN'n'CIN hypothesis can help to solve this long-standing problem by applying different thresholds for aneuploidy effects to each ontogeny period and to each tissue depending on the mitotic activity and pattern of the organization. To this end, regardless results of forthcoming experimental testing of this hypothesis, brain aging should be viewed as an extraordinarily complex biologic phenomenon that conceptually changes our thinking about time-scale of biological processes in neural cells as well as about causal relationship between somatic GIN/CIN and cellular homeostasis.

## Competing interests

The authors declare that they have no competing interests.

## Authors' contributions

YBY and IYI wrote the manuscript and SGV contributed original ideas and significant editorial input. All authors read and approved the final version of the manuscript.
